# The landscape overview of CD47-based immunotherapy for hematological malignancies

**DOI:** 10.1186/s40364-023-00456-x

**Published:** 2023-02-01

**Authors:** Hua Yang, Yang Xun, Hua You

**Affiliations:** 1 Department of Basic Medicine and Biomedical Engineering, School of Medicine, Foshan University, Foshan, Guangdong Province 528000 China; 2 Laboratory for Excellence in Systems Biomedicine of Pediatric Oncology, Department of Pediatric Hematology and Oncology, Children’s Hospital of Chongqing Medical University, Chongqing, 401122 China; 3 Chongqing Key Laboratory of Pediatrics, Ministry of Education Key Laboratory of Child Development and Disorders, National Clinical Research Center for Child Health and Disorders, Children’s Hospital of Chongqing Medical University, Chongqing, 401122 China

**Keywords:** CD47, SIRPα, Targeted therapies, Clinical trials, Immunotherapy

## Abstract

Extensive clinical and experimental evidence suggests that macrophages play a crucial role in cancer immunotherapy. Cluster of differentiation (CD) 47, which is found on both healthy and malignant cells, regulates macrophage-mediated phagocytosis by sending a "don't eat me" signal to the signal regulatory protein alpha (SIRPα) receptor. Increasing evidence demonstrates that blocking CD47 interaction with SIRPα can enhance cancer cell clearance by macrophages. Additionally, inhibition of CD47/SIRPα interaction can increase antigen cross-presentation, leading to T-cell priming and an activated adaptive antitumor immune response. Therefore, inhibiting CD47/SIRPα axis has a significant impact on tumor immunotherapy. Studies on CD47 monoclonal antibodies are at the forefront of research, and impressive results have been obtained. Nevertheless, hematotoxicity, especially anemia, has become the most common adverse effect of the CD47 monoclonal antibody. More specific targeted drugs (*i.e.*, bispecific antibodies, SIRPα/Fc fusion protein antibodies, and small-molecule inhibitors) have been developed to reduce hematotoxicity. Here, we review the present usage of CD47 antagonists for the treatment of lymphomas and hematologic neoplasms from the perspectives of structure, function, and clinical trials, including a comprehensive overview of the drugs in development.

## Background

### Structure, Expression, and function of CD47/SIRPα

Cancer treatment targeting immune checkpoints like programmed cell death receptor-1 (PD-1)/ Programmed cell death ligand-1 (PD-L1) has raised significant interest. Recently, pharmaceutical communities have shifted their attention to the development of new anti-cancer medicines that target innate immunity checkpoints, such as the immune checkpoint of macrophages: a cluster of differentiation (CD) 47/signal regulatory protein alpha (SIRPα) pathway.

**Fig. 1:**
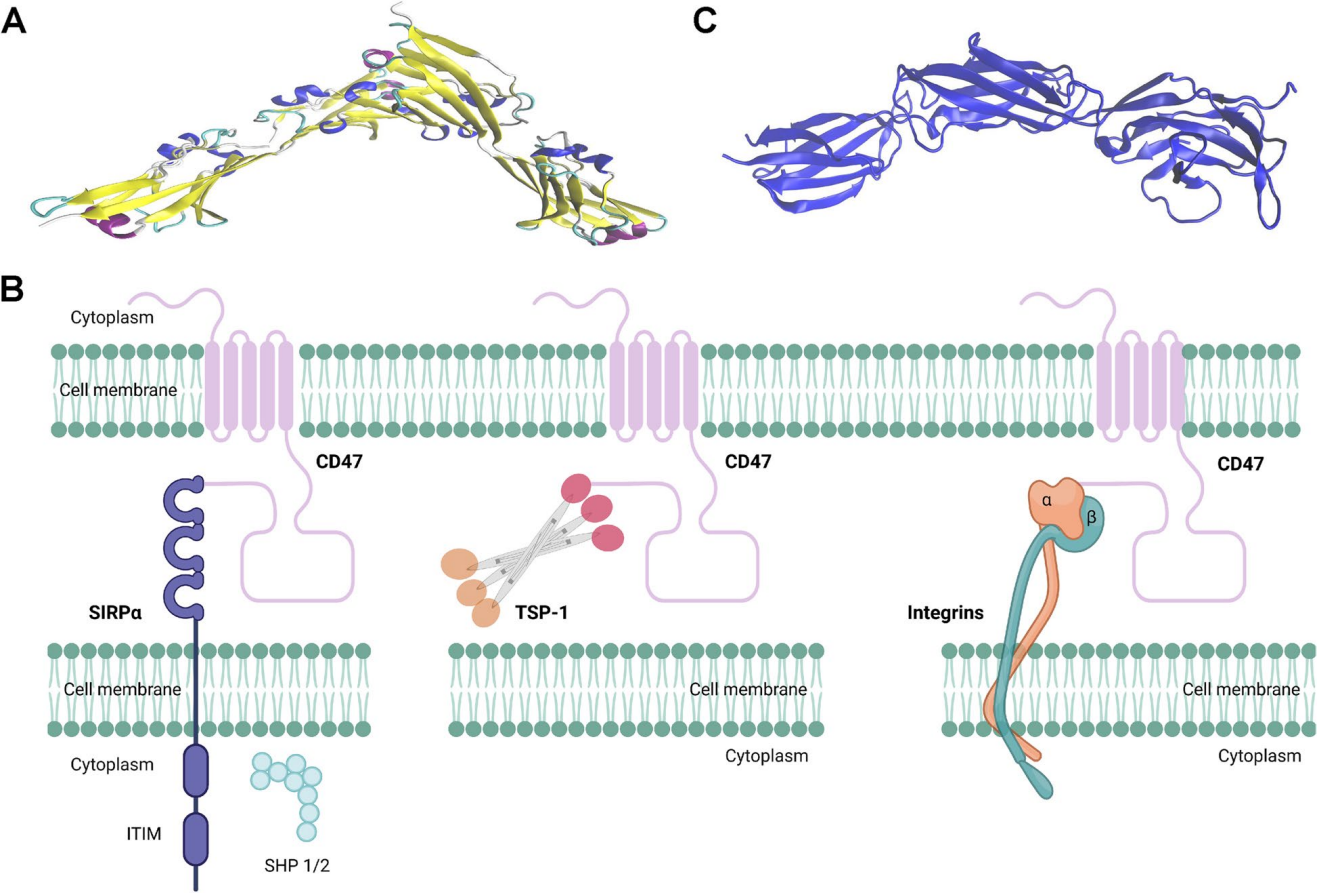
Overall structures of CD47, SIRPα and CD47 complex. **A** Structure of the ectodomain of human CD47 (PDB#2VSC). **B** Interaction of CD47 with three major ligands (SIRPα, TSP-1 and integrin). The figure was created by Biorender.com. **C** Complete extracellular region of human SIRPα (PDB#2WNG). The structures were reconstructed using VMD software. Abbreviations: CD47, cluster of differentiation 47; IgSF, immunoglobulin superfamily; ITIM, immunoreceptor tyrosine-based inhibitory motif; SHPS-1/2, protein tyrosine phosphatase substrate-1/2; SIRPα, signal-regulatory protein α; TSP-1: thrombospondin-1

CD47, formerly known as integrin-associated protein, is a 50 kDa plasma membrane molecule. CD47 is composed of an extracellular variable region that interacts with corresponding ligands (Fig. 1A), a transmembrane region comprised of highly hydrophobic transmembrane segments, and a hydrophilic carboxy-terminal intracellular region [1].

CD47 was firstly found as a transmembrane protein of red blood cells (RBCs). Current evidence indicates that CD47 is widely expressed in various normal human cell types, as well as in the membrane of different cancer cell types. In oncology study, CD47 was initially discovered as a tumor antigen in human ovarian cancer, and later was found overexpressed in various lymphomas and hematological tumors, such as non-Hodgkin's lymphomas (NHL) [2], T-cell lymphoma [3], acute myeloid leukemia (AML) and myelodysplastic syndrome (MDS) [4].

CD47 is capable of interacting with a variety of extracellular ligands, including SIRPα, thrombospondin-1 (TSP-1), integrins (α2β1, α4β1, α5β1, and α6β1), SIRPγ, CD36 and CD95, as well as with various intracellular ligands, such as the Gi proteins and Bcl-2/adenovirus E1B 19-kDa interacting protein 3 [1, 5]. Among these ligands, SIRPα, TSP-1 and integrins have been mostly studied (Fig. 1B).

**Fig. 2:**
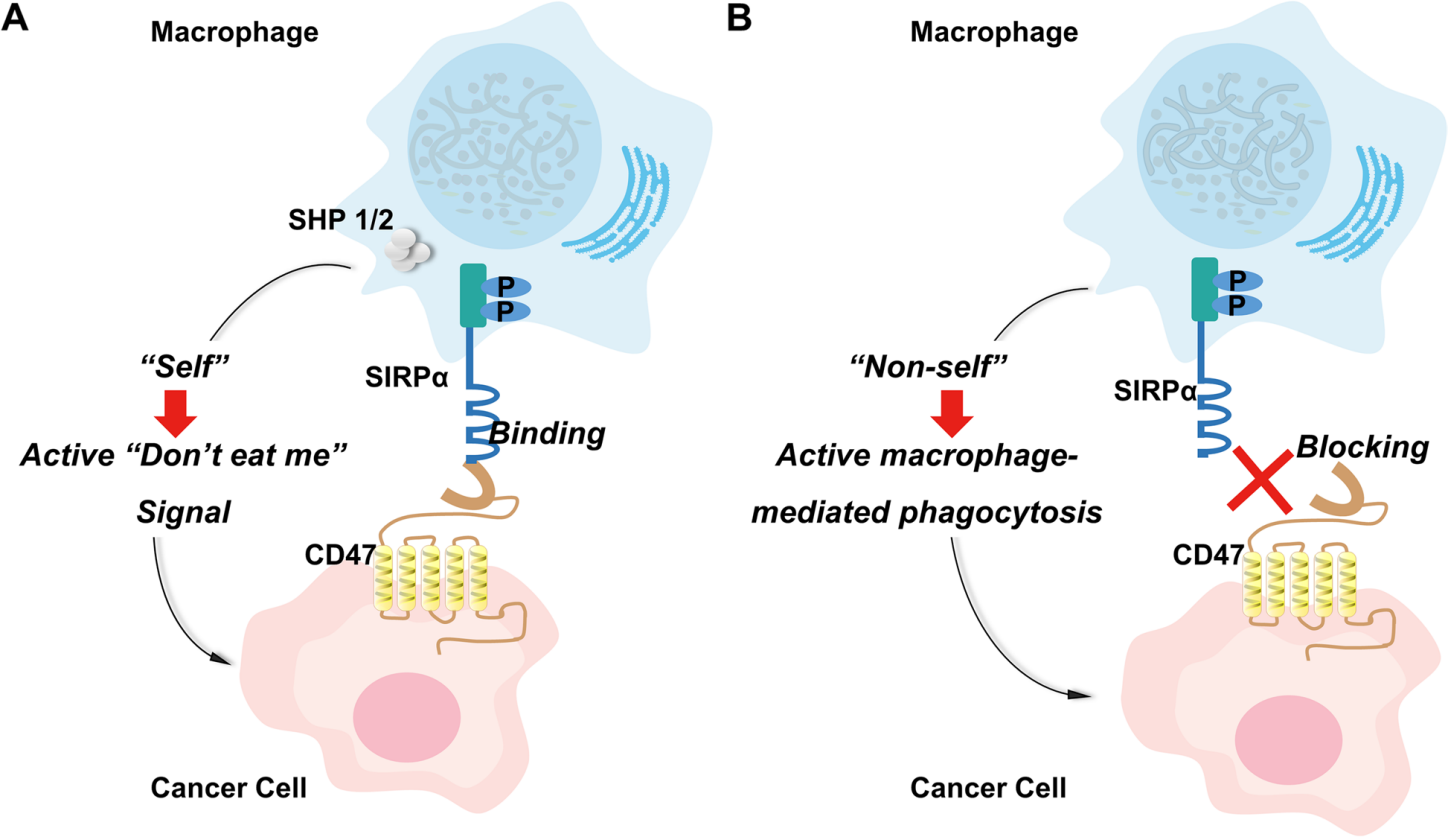
Macrophages distinguish between “self” or “non-self” by binding to SIRPα transmembrane protein on macrophage to form the CD47/SIRPα signaling complex. **A** CD47 expressed on cancer cell membrane binds to SIRPɑ on macrophage cell membrane to activate the “Don’t eat me” signal and block macrophage phagocytosis of cancer cells. **B** Blocking CD47-SIRPɑ interaction between cancer cell and macrophage induces phagocytosis by macrophage. Abbreviation: CD47, cluster of differentiation 47; SIRPα, signal-regulatory protein α

SIRPα is a member of the receptor family of signal regulatory proteins (SIRP) which involves five members (SIRPα, SIRPβ1, SIRPγ, SIRPβ2, and SIRPδ) encoded by a gene cluster located on chromosome 20p13 [6]. Among the family members, SIRPα is composed of an intracellular domain containing an immunoreceptor tyrosine-based inhibitor motif (ITIM), a transmembrane-spanning region, and three extracellular immunoglobulin superfamily domains (Fig. 1C). When CD47 binds to SIRPα, ITIM in the cytoplasmic tail of SIRPα is phosphorylated. Phosphatases including Src homology phosphatase (SHP)-1 and SHP-2, are then recruited and activated (Fig. 1C). CD47 is capable of distinguishing self or non-self cells via attaching to SIRPα which are mainly expressed on myeloid cells (monocytes, granulocytes, dendritic cells, and particularly macrophages [7–9]). When CD47 binds to SIRPα, the "don't eat me" signal is activated, inhibiting macrophage-mediated phagocytosis (Fig. 2) [10, 11].

TSP-1, which belongs to a thrombospondin family of five secreted glycoproteins, has a high binding affinity to CD47 at picomolar concentrations [12]. The interaction occurs via the C-terminal domain of TSP-1, and plays an important role in maintaining vascular tone, blood pressure, and modulating cardiac response [13]*.* In addition, several integrins are capable of interacting with CD47. For example, α5β1 is involved in chondrocyte mechanotransduction by binding to CD47 [14]. Dysregulation of αVβ3 and CD47 signaling leads to joint inflammation, cartilage destruction, and progression of osteoarthritis [15].

### Function and mechanism of CD47/SIRPα axis in tumor cells

CD47 expression is significantly elevated in leukemic cancer cells and supports these cells in evading phagocytosis by macrophages [16]. Numerous studies have indicated that CD47 is critical for treatment, prognosis, and diagnosis of a variety of malignancies, in which the most notable function of the CD47/SIRPα axis regards cancer therapy.

**Fig. 3:**
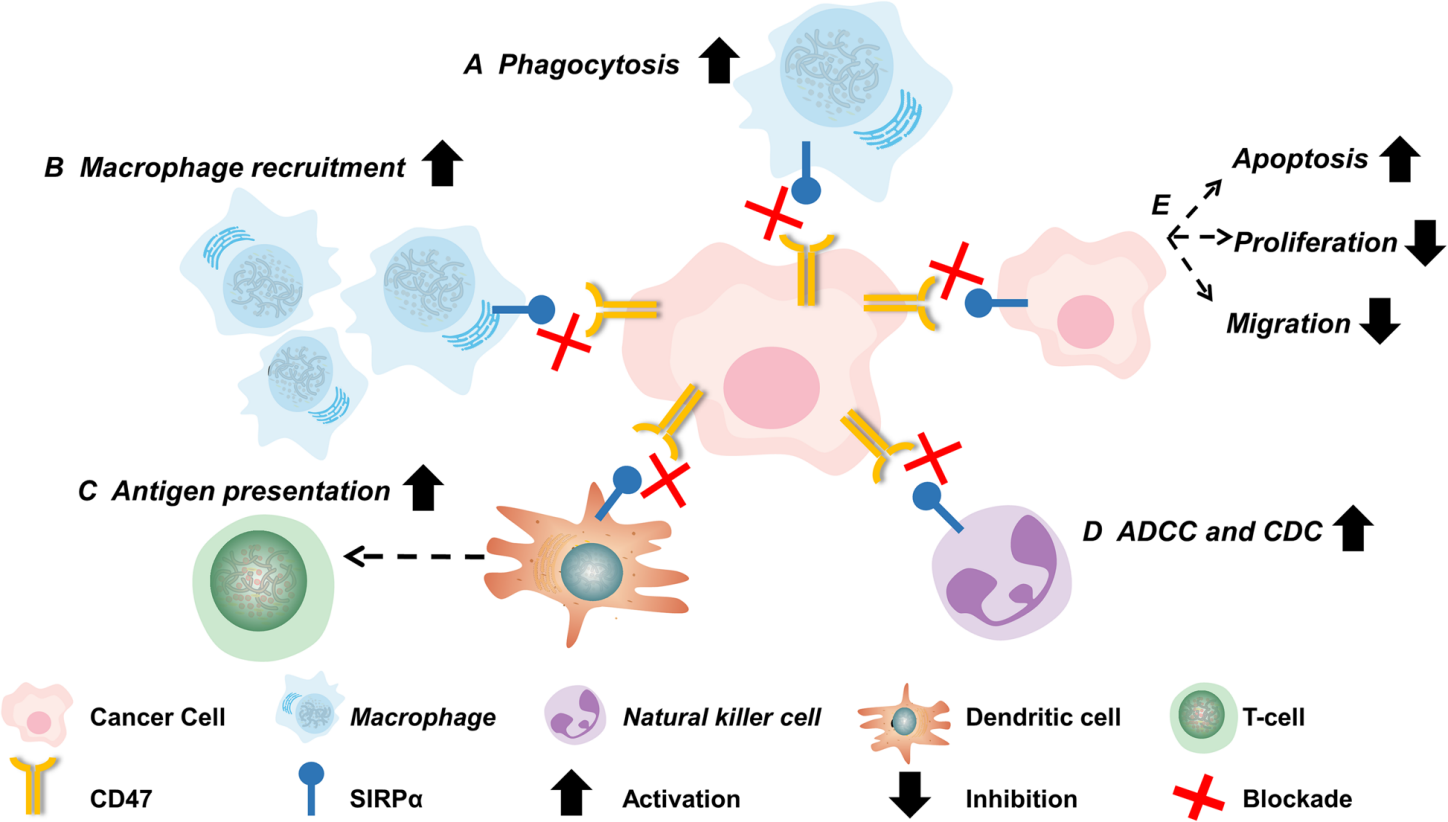
Inhibiting CD47/SIRPα axis regulates the fate of cancer cells**.** Inhibiting the CD47/SIRPα axis can (**A**) directly enhance phagocytosis of macrophages to tumor cells; **B** transform tumor-associated macrophages into an antitumor state and increase the recruitment of macrophages in tumors; **C** promote phagocytosis by dendritic cells and antigen presentation to CD8 + T-cells; (D) destroy tumor cells by natural killer cell-mediated ADCC and CDC; **E** increase tumor cell death, inhibit tumor cell proliferation, and prevent tumor cell migration. Abbreviation: CD47, cluster of differentiation 47; SIRPα, signal-regulatory protein α; ACDD: antibody-dependent cellular cytotoxicity; CDC: complement-dependent cytotoxicity

Recent research has shown that the CD47/SIRPα axis controls the destiny of tumor cells. Inhibiting the axis is able to enhance macrophage phagocytosis of tumor cells. So far five primary mechanisms of the CD47/SIRPα axis have been discovered (Fig. 3). Firstly, suppression of the CD47-SIRPα interaction results in macrophage phagocytosis of tumor cells. Full activation of macrophages requires two conditions: blockade of the CD47 "don't eat me" signal, and activation of the Fc receptor "eat me" signal. The presence of either can only provide a limited macrophage activation [17]. Secondly, blocking the CD47/SIRPα axis can transform tumor-associated macrophages into an antitumor state, and increase tumor macrophage recruitment [18, 19]. Thirdly, inhibition of the SIRPα/CD47 axis promotes phagocytosis by dendritic cells and subsequent antigen presentation to CD8 + T-cells, hence inducing an adaptive antitumor immune response [20, 21]. Moreover, CD47 antagonists destroy tumor cells utilizing natural killer cell-mediated antibody-dependent cytotoxicity (ADCC) and complement-dependent cytotoxicity (CDC) [22, 23]. Lastly, CD47 antagonists can promote tumor cell death [24, 25], reduce tumor cell proliferation [26–28], and prevent tumor cell migration [29, 30].

Additionally, CD47 can be used as a prognostic marker in a variety of cancers. High CD47 expression has been demonstrated to correlate with a poor outcome in AML [16], chronic myelogenous leukemia [31], NHL [32] and some solid tumors (*e.g.*, breast cancer, renal cell carcinoma, non-small cell lung cancer, thyroid cancer [33], etc.).

Aside from the implications mentioned above in tumor treatment and prognosis, CD47 has been implicated to aid the diagnosis of non-small cell lung cancer [34], renal cell tumors, and hematological tumors [33].

### Clinical development of CD47/SIRPα antibodies

In the past decade, patent applications for CD47 antagonists have increased steadily, reaching a high in 2019. The top five licensing authorities (the United States, the World Intellectual Property Organization, the European Patent Office, Japan, and China) hold a large number of CD47 antagonist patents (> 67% of all patents), in which numerous of them have entered clinical trials. Currently, CD47 antagonists are primarily classified into five categories: (1) CD47 monoclonal antibody; (2) CD47-targeted bispecific antibody; (3) SIRPα/Fc fusion protein antibody, (4) CD47 small-molecule inhibitor, and (5) CD47 antibody–drug conjugate. Since no clinical trials on CD47 small-molecule inhibitors or antibody–drug conjugates for hematologic malignancies are publicly available, only the first three categories will be discussed.

Recent clinical investigations employing CD47 monoclonal antibodies have produced excellent results. Anemia and thrombocytopenia are the most common adverse events (AEs) associated with CD47 monoclonal antibodies [35]. This is due to the fact that erythrocytes and platelets express CD47; CD47 monoclonal antibodies can attack them either through direct binding or through activation of NK cells and macrophages via Fc-mediated ADCC or CDC [36].

To reduce toxicity and improve treatment efficacy, researchers have developed CD47-targeted bispecific antibodies and the SIRPα/Fc fusion protein antibodies. The synergistic effect of bispecific antibodies targeting CD47 and other tumor antigens can potentially increase the safety and efficacy of treatment by targeting tumor cells preferentially [37].

SIRPα/Fc fusion protein antibodies are able to destroy CD47/SIRPα binding to decrease the CD47 "don't eat me" signal and generate an activating prophagocytic signal via Fc receptors [3, 32, 38]. Since SIRPα is not expressed on human blood cells, SIRPα/Fc fusion protein antibodies display negligible binding to RBCs or blood platelets, distinguishing them from anti-CD47 monoclonal antibodies [39].

The CD47/SIRPα axis has been identified as a potential future immunotherapeutic target for hematological malignancies. Here, we provide a summary of CD47 antagonist clinical research frontiers in treating lymphomas and hematological malignancies. All clinical research and trial information was gathered from the PubMed database, Researchgate database, the US national clinical trials registry (NCT) system www.clinicaltrials.gov and the China drug trials registry (CTR) system www.chinadrugtrials.org.cn.

## CD47 antagonist in lymphomas treatment

Currently, twenty-three CD47 antagonists are available in clinical trials for the treatment of lymphomas (Table 1). A list of CD47 antagonists with published clinical data is shown (Table 2).

**Table 1:** CD47 antagonists currently entering clinical trials for treatment of lymphomas

Drug Name	Other Name	Companies	Target-based Actions	Drug Classification	Indications	US Highest Phase	Chinese Highest Phase	NCT	CTR
CC-90002	INBRX-103	Bristol-Myers Squibb Company	CD47 antagonist	anti-CD47 monoclonal antibody	Non-Hodgkin lymphoma	Phase I	/	NCT02641002	/
GenSci-059	GenSci-059	GeneScience Pharmaceuticals Co Ltd	CD47 antagonist	anti-CD47 monoclonal antibody	Lymphoma	Phase I	/	NCT05221385	/
IMC-002	3D-197	ImmuneOncia Therapeutics LLC	CD47 antagonist	anti-CD47 monoclonal antibody	Lymphoma	Phase I	/	NCT04306224	/
Lemzoparlimab	TJ011133; TJC4	I-Mab Bio-Tech (Tianjin) Co., Ltd	CD47 antagonist	anti-CD47 monoclonal antibody	Lymphoma	Phase I	/	NCT03934814	/
					CD20 positive lymphoma	/	Phase I	/	CTR20210313
Letaplimab	IBI-188	Innovent Biologics Inc	CD47 antagonist	anti-CD47 monoclonal antibody	Lymphoma	Phase I	Phase I	NCT03717103, NCT03763149, NCT04861948	CTR20182140
Ligufalimab	AK117	Akeso Biopharma Inc	CD47 antagonist	anti-CD47 monoclonal antibody	Lymphoma	Phase I	Phase I	NCT04728334, NCT04349969	CTR20202684
Magrolimab	Hu5F9-G4	Gilead Sciences	CD47 antagonist	anti-CD47 monoclonal antibody	Non-Hodgkin lymphoma	Phase II	/	NCT03527147 NCT02953509	/
					Classic Hodgkin lymphoma	Phase II	/	NCT04788043	/
					T-Cell lymphoma	Phase II	/	NCT04541017	/
MIL-95	CM-312	KeyMed Biosciences Co Ltd	CD47 antagonist	anti-CD47 monoclonal antibody	Lymphoma	Phase I	Phase I	NCT04651348	CTR20201108
SHR-1603	SHR-1603	Jiangsu Hengrui Medicine Co Ltd	CD47 antagonist	anti-CD47 monoclonal antibody	Lymphoma	Phase I	Phase I	NCT03722186	CTR20181964
ZL-1201	ZL-1201	Zai Lab Limited	CD47 antagonist	anti-CD47 monoclonal antibody	Lymphoma	Phase I	Phase I	NCT04257617	CTR20210973
BAT-7104	BAT-7101	Bio-Thera Solutions Ltd	CD47 antagonist; Programmed cell death ligand 1 inhibitor	bispecific antibody	Lymphoma	Phase I		/	CTR20220098
HX-009	HX-009–5	HanX Biopharmaceuticals Inc	CD47 antagonist; Programmed cell death protein 1 inhibitor	bispecific antibody	Lymphoma	Phase II	Phase II	NCT05189093	CTR20213391
IBI-322	IBI-322	Innovent Biologics Inc	CD47 antagonist; Programmed cell death ligand 1 inhibitor	bispecific antibody	Lymphoma	Phase I	/	NCT04338659	/
IMM-0306	IMM-0306	ImmuneOnco Biopharm Co Ltd	B-lymphocyte antigen CD20 modulator; CD47 antagonist; Immunoglobulin gamma Fc receptor agonist	bispecific antibody	CD20-positive B-cell non-Hodgkin's lymphoma	Phase I	/	NCT04746131	CTR20192612
JMT-601	CPO-107	Shanghai JMT-Bio Inc	B-lymphocyte antigen CD20 modulator; CD47 antagonist	bispecific antibody	CD20 positive B-cell non-Hodgkin's lymphoma	Phase II	Phase II	NCT04853329	CTR20211365
SG-12473	SG-12473	Hangzhou Sumgen Biotechnology Co Ltd	CD47 antagonist; Programmed cell death ligand 1 inhibitor	bispecific antibody	Lymphoma	/	Phase I	/	CTR20211029
TG-1801	NI-1701	TG Therapeutics Inc	B-lymphocyte antigen CD19 modulator; CD47 antagonist; Immunoglobulin Fc receptor agonist	bispecific antibody	B-Cell Lymphoma	Phase I	/	NCT03804996, NCT04806035	/
XL-114	AU-341; AU7R-104; AUR-104	Exelixis Inc	Bcl-10 protein modulator; CD47 antagonist; Caspase recruitment domain protein 11 modulator; Epidermal fatty acid binding protein inhibitor	bispecific antibody	Non-Hodgkin lymphoma	Phase I	/	NCT05144347	/
Evorpacept	ALX-148	ALX Oncology	SIRPα-Fc fusion protein,SIRPα/CD47 blocker	SIRPα/Fc fusion protein antibody	B-cell Non-Hodgkin Lymphoma	Phase II	/	NCT05025800, NCT03013218	/
IMM-01	IMM-01	ImmuneOnco Biopharm Co Ltd	CD47 antagonist	SIRPα/Fc fusion protein antibody	Hodgkin's lymphoma, B-cell non-Hodgkin lymphoma; NK/T-cell lymphoma	/	Phase II	/	CTR20212227
IMM-01	IMM-01	ImmuneOnco Biopharm Co Ltd	CD47 antagonist	SIRPα/Fc fusion protein antibody	Lymphoma	/	Phase II	/	CTR20191531
TTI-621	TTI-621	Trillium Therapeutics Inc	CD47 antagonist; Immunoglobulin gamma Fc receptor agonist	SIRPα/Fc fusion protein antibody	Mycosis fungoides	Phase I	/	NCT02890368	/
TTI-622	TTI-622	Trillium Therapeutics Inc	CD47 antagonist; Immunoglobulin gamma Fc receptor agonist	SIRPα/Fc fusion protein antibody	Lymphoma	Phase I	/	NCT03530683	/
RRx-001	RRx-001	EpiCentrx Inc	CD47 antagonist; DNA methyltransferase inhibitor; Epigen modulator; Programmed cell death ligand 1 inhibitor; Tyrosine phosphatase substrate 1 inhibitor	small-molecule inhibitor	Lymphoma	Phase I	/	NCT01359982, NCT02096341, NCT02518958	/

**Table 2:** Published results of clinical trials on the use of CD47 antagonists in lymphomas

Drug Name	NCT Number	Sponsor	Protocol	Indication Detail	Enroll-ment	Age/Race/Healthy Volunteers	Phase	Status	Start Date	Design	Endpoint Types	Efficacy Results	Ref
ALX148	NCT03013218	ALX Oncology	ALX148	advanced malignancy and non-Hodgkin lymphoma	25 (ALX148 monotherapy per week); 11 (ALX148 monotherapy at the highest dose of 30 mg/kg once every other week)	Adults (18 and over)	Phase I	No longer recruiting	2017–01-06	Interventional; Treatment; Non-Randomized; Open Label; Single Group Assignment	Safety; Efficacy; Pharmacokinetics; Pharmacodynamics	SD: 27% (ALX148 monotherapy per week), 18% (ALX148 monotherapy at the highest dose of 30 mg/kg once every other week)	[40]
ALX148	NCT03013218	ALX Oncology	ALX148; rituximab	Patients with relapsed or refractory CD20-positive B-cell NHL	33	Adults (18 and over)	Phase I	No longer recruiting	2017–01-06	Interventional; Treatment; Non-Randomized; Open Label; Single Group Assignment	Safety; Efficacy; Pharmacokinetics; Pharmacodynamics	ORR: 45%	[40]
TJ011133	NCT03934814	I-Mab Bio-Tech (Tianjin) Co., Ltd	lemzoparlimab; rituximab	R/R patients with CD20 positive Non-Hodgkin’s Lymphoma	8	Adults (18 and over)	Phase I	Recruiting	2019–04-16	Interventional; Treatment; Non-Randomized; Open Label; Single Group Assignment	Safety; Efficacy; Pharmacokinetics; Pharmacodynamics	ORR: 57%	[41]
IBI-188	NCT03763149	Innovent Biologics Inc	letaplimab	advanced/refractory solid tumors or lymphoma	20	Adults (18 and over)	Phase I	Completed	2019–02-19	Interventional; Treatment; Open Label; Single Group Assignment	Safety; Efficacy; Pharmacokinetics; Pharmacodynamics	/	[42]
Hu5F9-G4	NCT02953509	Forty Seven Inc; Gilead Sciences Inc	Hu5F9-G4; rituximab	Relapsed/Refractory B-cell Non-Hodgkin's Lymphoma	22	Adults (18 and over)	Phase I/II	No longer recruiting	2016–11-21	Interventional; Treatment; Non-Randomized; Open Label; Single Group Assignment	Safety; Efficacy; Pharmacokinetics; Pharmacodynamics	ORR: 50%	[43]
TTI-621	NCT02663518	Trillium Therapeutics Inc	TTI-621	T-Cell Lymphoma	64	Adults (18 and over)	Phase I	No longer recruiting	2016–01-01	Interventional; Treatment; Non-Randomized; Open Label; Parallel Assignment	Safety; Efficacy; Pharmacokinetics; Pharmacodynamics	ORR (CTCL): 19%; ORR (PTCL): 18%	[44]
TTI-621	NCT02663518	Trillium Therapeutics Inc	1. TTI-621; rituximab 2. TTI-621	DLBCL	35	Adults (18 and over)	Phase I	No longer recruiting	2016–01-01	Interventional; Treatment; Non-Randomized; Open Label; Parallel Assignment	Safety; Efficacy; Pharmacokinetics; Pharmacodynamics	ORR: 21% vs 29% (TTI-621 plus rituximab vs TTI-621 monotherapy)	[44]
TTI-621	NCT02890368	Trillium Therapeutics Inc	TTI-621	Relapsed/Refractory Mycosis Fungoides and Sézary Syndrome	35	Adults (18 and over)	Phase I	Terminated	2016–09-01	Interventional; Treatment; Non-Randomized; Open Label; Parallel Assignment	Safety; Efficacy; Pharmacokinetics; Pharmacodynamics	≥ 50% reduction in CAILS score: 34%	[45]
TTI-622	NCT03530683	Trillium Therapeutics Inc	TTI-622	patients with advanced relapsed or refractory lymphoma	42	Adults (18 and over)	Phase I	Recruiting	2018–05-01	Interventional; Treatment; Non-Randomized; Open Label	Safety; Efficacy; Pharmacokinetics; Pharmacodynamics	ORR: 33%	[46, 47]

### CD47 monoclonal antibodies

#### CC-90002

CC-90002 is the first generation of humanized IgG4 anti-CD47 antibody entering clinical research. CC-90002 plus rituximab (an anti-CD20 monoclonal antibody) demonstrated tolerability and modest clinical activity in the heavily pretreated relapsed/refractory (R/R) NHL patients [48]. AEs were predominantly Grade 1/2, and the most frequent Grade 3/4 AEs were neutropenia (38%) and thrombocytopenia (21%). Twenty-four subjects were treated, one subject attained a stable complete response rate (CR), two subjects achieved partial response (PR), and three subjects achieved stable disease (SD). Although the fact that the current data suggest further investigation of CC-90002 in conjunction with rituximab for the treatment of NHL, no Phase II clinical studies have been conducted.

#### Hu5F9-G4

Hu5F9-G4 (5F9), also known as magrolimab, is a humanized monoclonal IgG4 antibody that was independently developed by Stanford University Forty Seven (Stanford, CA, USA) [49]. 5F9 not only inhibits CD47 signaling, which increases macrophage phagocytosis, but also stimulates ADCC [50]. Studies have demonstrated that 5F9 was more effective in combination with other antibodies than alone in the treatment of non-Hodgkin lymphoma [1, 43, 51, 52].

A phase Ib study (NCT02953509) assessed the safety and efficacy of 5F9 in combination with rituximab in 22 patients with R/R lymphoma (15 with DLBCL and 7 with FL) [43]. According to this study, CD47 receptors were present on more than 90% of peripheral blood cells. The overall response rate (ORR) and complete response rate (CR) were 50% and 36%, respectively. ORR and CR rates for DLBCL patients were 40% (6/15) and 33% (5/15), respectively. The ORR and CR for patients with a diagnosis of FL were 71% (5/7) and 43% (3/7), respectively. Anemia was the most prevalent AE, occurring in around 42% of patients, while all were manageable. To treat anemia, priming doses of 1 mg/kg 5F9 and maintenance doses of 30 mg/kg every week (QW) were administered. This study demonstrates that the combination of 5F9 and rituximab is effective in patients with R/R DLBCL or FL without a considerable risk of AEs.

In 2018, the Food and Drug Administration (FDA) approved 5F9 for the treatment of two types of R/R B-cell NHL (DLBCL and FL). Ongoing clinical trials involve the combination of 5F9 and rituximab (NCT03527147), the BTK inhibitor acalabrutinib (NCT03527147), mogamulizumab (NCT04541017), and pabolizumab (NCT04788043).

#### TJ011133 (TJC4, lemzoparlimab)

TJ011133 (TJC4, also known as lemzoparlimab) is a therapeutic anti-CD47 IgG4 antibody of the next generation investigated by I-Mab Biopharma (Beijing, China). TJ011133 has a unique binding epitope and an RBC/platelet sparing characteristic; therefore it does not produce substantial hematologic toxicity and does not require priming doses like 5F9 [49].

Eight R/R patients with CD20-positive NHL who had received at least two prior lines of therapy were included in a Phase Ib research (NCT03934814) [41]. TJ011133 was administered intravenously at doses of 20 or 30 mg/kg weekly with rituximab. All treatment-related AEs were grade 1 or 2 except for one patient who reported Grade 3 treatment-related AEs. CD47 receptor occupancy was 80% and 90% in those who received 20 mg/kg and 30 mg/kg of TJ011133, respectively. The assessable efficacy of seven individuals showed an ORR of 57% (three CR, one PR and the rest SD). The combo therapy demonstrated therapeutic effectiveness for individuals with R/R NHL. In addition, no priming dose was required for TJ011133 in this clinical research.

#### AK117 (Ligufalimab)

AK117 is a novel humanized IgG4 monoclonal antibody with a unique structure. Due to the unique conformation of AK117/CD47 complex, AK117 has no hemagglutination effect. A phase I clinical trial of AK117 revealed that it was well-tolerated up to 45 mg/kg per week in participants with no dose-limiting toxicity events and no hematological treatment-related AEs. As a result, AK117 does not need a lower 'priming' dose to prevent anemia. The CD47 receptor occupancy of AK117 on T cells in peripheral blood of participants reached and sustained at 100% at a dose of 3 mg/kg alone, with complete and lasting receptor occupancy in peripheral blood found at ≥ 10 mg/kg. AK117 has an excellent safety profile in clinical applications. A series of clinical trials of AK117 alone or in combination with multiple agents (*e.g.*, rituximab) are ongoing for the treatment of a variety of hematologic malignancies [53].

### CD47-targeted bispecific antibodies

#### CD47/CD20-targeted bispecific antibodies

##### IMM0306

Both in vivo and in vitro experiments confirmed that comparing with CD47 monoclonal antibodies, anti-CD47/CD20 bispecific antibodies showed better binding preference to tumor cells and more potent anti-lymphoma activity [54, 55]. Researchers suggest that anti-CD47/CD20 bispecific antibodies might be viable candidates for clinical trials, in which IMM0306 was the first of these to report preclinical results.

IMM0306 is a fusion protein of CD20 monoclonal antibody with CD47 binding domain of SIRPα. It exerts excellent cancer killing efficacy by activating both macrophages and NK cells via blockade of CD47-SIRPα interaction and FcɣR engagement by simultaneously binding to CD47 and CD20 of B cells. Extensive in vitro analysis revealed that IMM0306 had a strong affinity for a variety of hematologic malignant cells. Concerning the usage safety, IMM0306 has no binding activity on human RBCs. IMM0306 showed stronger ADCC activity and lower CDC activity in various hematologic malignancy cells when compared to rituximab, possibly due to the fact that the Fc segment of the recombinant protein in IMM0306 is IgG1. Application of IMM0306 in treating tumor-implanted SCID mice significantly inhibited tumor growth. Furthermore, in a lymphoma orthotopic model, IMM0306 paired with lenalidomide outperformed any single medicine or rituximab combination with lenalidomide in terms of therapeutic impact [17]. IMM0306 is being tested in two phase I clinical trials in patients with R/R CD20-positive B-cell NHL (NCT04746131 and CTR20192612).

#### CD47/CD19-targeted bispecific antibody

##### TG-1801 (NI-1701)

TG-1801 is an investigational first-in-class, bispecific anti-CD47/CD19 monoclonal antibody. TG-1801 has exhibited a potent capability to induce ADCP and ADCC in malignant B-cell lines and primary tumor B-cells from patients with ALL, CLL, and different subtypes of NHL in preclinical studies [56]. TG-1801 combined with rituximab was observed to have a stronger tumor-killing synergy than applying rituximab alone [36]. TG-1801 was also confirmed to be compatible with rituximab [57] and umbalixib (a phosphatidylinositol 3-kinase δ inhibitor) [57, 58] for the treatment of B-cell NHL and CLL.

TG-1801 is currently being evaluated in two phase I trials (NCT03804996 and NCT04806035) to evaluate its safety and efficacy in treating patients with B-cell lymphoma and CLL. No clinical data on TG-1801 have been reported so far.

### SIRPα/Fc fusion protein antibodies

#### TTI-621 (SIRPα-IgG1 Fc)

TTI-621 is a fusion protein developed from the CD47 binding domain of human SIRPα linked to the Fc region of human IgG1. It is intended to improve phagocytosis and anti-cancer activity of macrophages by preventing CD47-SIRPα interaction between malignant cells and macrophages through Fc receptors engagement [45]. In preclinical studies, TTI-621 was shown to enhance macrophage phagocytosis of various malignant cells and decreased the growth of AML and B-cell lymphoma in xenografts. Besides, TTI-621 also displayed low binding affinity to human erythrocytes [3].

NCT02663518 is the First-in-human (FIH) phase I study in patients with R/R lymphoma [3]. This study aimed to evaluate the safety and efficacy of TTI-621 as a monotherapy or combination with rituximab or nivolumab. The MTD of the TTI-621 single-drug and the combined groups were 0.2 and 0.1 mg/kg, respectively. No death happened as a result of treatment-related adverse events, and only 37% of the patients experienced adverse events (AEs) of grade 3 or higher. Twenty percent of patients experienced thrombocytopenia, which was reversible and often cleared within one week. At the highest dose evaluated (0.5 mg/kg), the systemic exposure of TTI-621 showed dose-dependent without a plateau. The receptor occupancy rate was 34% and 66% at 0.2 and 0.5 mg/kg groups, respectively. Furthermore, single TTI-621 dosages up to 0.5 mg/kg did not increase the incidence of thrombocytopenia as compared to the 0.2 mg/kg group. The ORRs for TTI-621 monotherapy and TTI-621 plus rituximab for DLBCL were 29% (2/7) and 21% (5/24), respectively. The ORR for TTI-621 monotherapy in T-cell NHL was 25% (8/32). Updated results from clinical study NCT02663518 showed that TTI-621 demonstrated ORR in 14/71 (20%) NHL patients, including those with cutaneous T-cell lymphoma (*n* = 42, one CR, seven PR), peripheral T-cell lymphoma (*n* = 22, two CR, two PR), and DLBCL (*n*= 7, one CR, one PR) [44].

Another phase I clinical trial (NCT02890368) confirmed that topically administered TTI-621 was tolerated and had systemic and local antitumor activity in patients with R/R fungal disease and Sezary syndrome [45]. TTI621 was administered intralesionally to 35 individuals. The maximum assessed dosing regimen in this trial was 10 mg, and the MTD was not met. Although 25 (71%) individuals experienced treatment-related adverse events, they were all grade 1 or 2. During the trial, the Composite Assessment of Index Lesion Severity (CAILS) scores were reduced in 26 (90%) of the 29 patients, with CAILS score reductions of 50% or more in 10 (34%) of the patients. The median time to respond for the nine patients with a CAILS response on TTI-621 monotherapy was 45 days (95% CI 17–66). Even single injections reduced CAILs scores, and lesions adjacent to the injection site in eight patients showed decreases. Continuing monotherapy with TTI-621 induced further reductions in CAILS scores up to 100%. Nevertheless, the efficacy of TTI-621 in combination with pegylated interferon alpha-2a or a PD-1/PD-L1 inhibitor appearred poor in the trial.

In conclusion, TTI-621 is well-tolerated and can be used as monotherapy in patients with R/R NHL and combined with rituximab in patients with R/R B cell- NHL.

#### TTI-622 (SIRPα/IgG4 Fc)

Like TTI-621, TTI-622 is a soluble SIRPα/Fc variant protein containing an IgG4 Fc tail. TTI-622 resulted in a statistically significant tumor growth reduction and improved survival in both early and delayed treatment in DLBCL xenograft tumor model. TTI-622 monotherapy showed partial tumor growth inhibition in Burkitt lymphoma and in MM xenograft models. Additionally, the therapeutic effect was further enhanced when TTI-622 was combined with daratumumab (an anti-CD38 antibody). Importantly, TTI-622 does not bind to human RBCs [46, 47].

The data above supports the clinical evaluation of TTI-622 in combination with other antitumor antibodies in hematological malignancies patients. There is an ongoing multicenter, phase I dose-escalation and expansion trial of TTI-622 (NCT03530683). TTI-622 was given to 42 individuals with R/R lymphoma at weekly doses ranging from 0.05 to 18 mg/kg. Treatment-related AEs have occurred in 20 (47%) patients, and most AEs have been Grade 1 or 2 and reversible. Related AEs of Grade ≥ 3 intensity has occurred in 7 (16.7%) patients. No significant dose relationship was observed between the AEs. Preliminary pharmacokinetics data indicated a dose-dependent increase in exposure after single and repeated TTI-622 infusions. According to pharmacodynamics results, end-of-infusion CD47 receptor occupancy is greater than 60% at 2 mg/kg doses. Objective responses occurred in 33% (9/27) of response-evaluable patients at doses ranging from 0.8 to 18 mg/kg. Of the nine patients, two achieved CR (one in DLBCL and one in cutaneous T-cell lymphoma—mycosis fungoides), and seven achieved PR (two in cutaneous T-cell lymphoma, two in peripheral T-cell lymphoma, two in DLBCL, and one in FL) [46, 47].

Based on these preliminary results, TTI-622 is currently being evaluated in various combination regimens in ongoing studies.

#### ALX148

ALX148 (also known as evorpacept) is a new CD47-blocking molecule produced by connecting a modified SIRPα D1 domain to an inactive human IgG1 Fc. ALX148 exhibits a high affinity for CD47 in many species, inhibits wild-type SIRPα binding, and promotes tumor cell phagocytosis by macrophages. ALX148 had little effect on normal blood cells in experiments with rodents and NHPs. In addition, ALX148 enhanced anti-cancer activity of obinutuzumab and rituximab (both anti-CD20 antibodies) in carcinogenesis murine xenograft models employing human lymphoma (Z138 and Raji) cell lines [59].

Preliminary activity in combination with rituximab was observed in R/R CD20-positive B-cell NHL patients with no curative treatment. Thirty-three patients were enrolled in a phase I clinical trial (NCT03013218) aiming to evaluate the effects of different dosages of ALX148 in conjunction with rituximab. A total of eleven patients received ALX148 (15 mg/kg QW) in combination with rituximab and 63.6% of them achieved ORR (three CR and four PR). In the fully enrolled safety cohorts, no Dose-limiting toxicities (DLTs) have been reported, and the MTD of ALX148 combined with rituximab had yet to be reached. There have been no reports of dose-limiting toxicities (DLTs), and the MTD of ALX148 combined with rituximab had not yet been reached. No treatment-related fatality was documented, and 16/33 patients experienced low-grade AEs [40].

#### IMM01

IMM01 is a recombinant human SIRPα IgG1 fusion protein that has strong dual-functional anti-tumor activity through phagocytosis with improved potency upon N-glycosylation removal [10]. IMM01 exhibits promising preclinical characteristics regarding its link between receptor occupancy, tumor exposure and efficacy. Moreover, IMM01 shows a unique property of weak human erythrocyte conjugation to avoid severe hemolysis [60].

Preliminary results of an FIH phase I study (CTR1900024904) in patients with R/R lymphoma revealed that 14 patients with R/R lymphoma who had failed standard therapies received IMM01 monotherapy [60]. No DLTs were detected at a dose up to 1 mg/kg. Most treatment-related side effects were grade 1 or 2, but one patient had a grade 3 temporary platelet count decrease after two hours of drug implication, which returned to baseline 24 to 48 h after the initial infusion. The ORR was 14.3% (one CR and one PR), and two patients had verified SD. According to the research, IMM01 demonstrated an outstanding preclinical safety, tolerability, and prospective antitumor effectiveness at dose up to 1.0 mg/kg.

## CD47 antagonist in the treatment of hematological tumors

There are currently 17 CD47 antagonists being tested for the treatment of hematological tumors in clinical trials (Table 3). CD47 antagonists with published clinical data are listed in Table 4.

**Table 3:** CD47 antagonists currently entering clinical trials for treatment of hematological tumors

Drug Name	Other Name	Companies	Target-based Actions	Drug Classification	Indications	US Highest Phase	Chinese Highest Phase	NCT	CTR
AO-176	AO-104	Arch Oncology	CD47 antagonist	anti-CD47 monoclonal antibody	Multiple myeloma	Phase II	/	NCT04445701	/
CC-90002	INBRX-103	Celgene Corp	CD47 antagonist	anti-CD47 monoclonal antibody	Acute myeloid leukaemia	Phase I	/	NCT02367196	/
					Myelodysplastic syndrome	Phase I	/	NCT02367196	/
GenSci-059	GenSci-059	GeneScience Pharmaceuticals Co Ltd	CD47 antagonist	anti-CD47 monoclonal antibody	Acute myeloid leukaemia	Phase I	/	NCT05263271	/
					Myelodysplastic syndrome	Phase I		NCT05263271	/
Lemzoparlimab	TJ011133; TJC4	I-Mab Bio-Tech (Tianjin) Co., Ltd	CD47 antagonist	anti-CD47 monoclonal antibody	Acute myeloid leukaemia	Phase II	Phase II	NCT04202003, NCT04912063	CTR20210555, CTR20192522
					Myelodysplastic syndrome	Phase II	Phase III	NCT04202003, NCT04912063	CTR20210555, CTR20192522, CTR20230090
					Multiple Myeloma	Phase I	/	NCT04895410	/
Letaplimab	IBI-188	Innovent Biologics Inc	CD47 antagonist	anti-CD47 monoclonal antibody	Acute myeloid leukaemia	Phase II	Phase II	NCT04485052	CTR20200938
					Myelodysplastic syndrome	Phase I	Phase III	NCT04511975, NCT04485065	CTR20201039
Ligufalimab	AK117	Akeso Biopharma Inc	CD47 antagonist	anti-CD47 monoclonal antibody	Acute myeloid leukaemia	Phase II	Phase II	NCT04980885	CTR20211305
					Myelodysplastic syndrome	Phase II	Phase II	NCT04900350	CTR20210825
Magrolimab	Hu5F9-G4	Gilead Sciences	CD47 antagonist	anti-CD47 monoclonal antibody	Myelodysplastic syndrome	Phase III	/	NCT04313881, NCT03527147	/
					Acute myeloid leukaemia	Phase III	/	NCT05263271, NCT04435691, NCT03248479, NCT03922477, NCT02678338	/
					Myelodysplastic syndrome	Phase II	/	NCT05263271, NCT03248479, NCT02678338	/
ZL-1201	ZL-1201	Zai Lab Limited	CD47 antagonist	anti-CD47 monoclonal antibody	hematologic malignancies	Phase I	Phase I	NCT04257617	CTR20210973
IBI-322	IBI-322	Innovent Biologics Inc	CD47 antagonist; Programmed cell death ligand 1 inhibitor	bispecific antibody	Hematological neoplasm	Phase I	Phase I	NCT04795128	CTR20210385
					Myeloid leukemia	Phase I	Phase I	NCT05148442	CTR20213120
SIRPa-Fc-CD40L	SL-172154	Shattuck Labs Inc	CD40 ligand receptor agonist; CD47 antagonist	bispecific antibody	Acute myeloid leukaemia	Phase I		NCT05275439	/
					Myelodysplastic syndrome	Phase I		NCT05275439	/
TG-1801	NI-1701	TG Therapeutics Inc	B-lymphocyte antigen CD19 modulator; CD47 antagonist; Immunoglobulin Fc receptor agonist	bispecific antibody	Chronic lymphocytic leukemia	Phase I	/	NCT04806035	/
XL-114	AU-341; AU7R-104; AUR-104	Exelixis Inc	Bcl-10 protein modulator; CD47 antagonist; Caspase recruitment domain protein 11 modulator; Epidermal fatty acid binding protein inhibitor	bispecific antibody	Chronic lymphocytic leukemia	Phase I	/	NCT05144347	/
DSP-107	DSP-107	KAHR Medical Ltd	CD47 antagonist; CDw137 agonist	SIRPα/Fc fusion protein antibody	Acute myeloid leukaemia	Phase I	/	NCT04937166	/
					Myelodysplastic syndrome	Phase I		NCT04937166	/
					Chronic myelomonocytic leukemia	Phase I	/	NCT04937166	/
Evorpacept	ALX-148	ALX Oncology	SIRPα-Fc fusion protein,SIRPα/CD47 blocker	SIRPα/Fc fusion protein antibody	Acute myeloid leukaemia	Phase II	/	NCT04755244	/
					Myelodysplastic syndrome	Phase II	/	NCT04417517	/
IMM-01	IMM-01	ImmuneOnco Biopharm Co Ltd	CD47 antagonist	SIRPα/Fc fusion protein antibody	Acute myeloid leukaemia	Phase II	Phase II	NCT05140811	CTR20212227, CTR20212519
					Myelodysplastic syndrome	Phase II	Phase II	NCT05140811	CTR20212227, CTR20212519
					Multiple myeloma	/	Phase II	/	CTR20212227
TTI-621	TTI-621	Trillium Therapeutics Inc	CD47 antagonist; Immunoglobulin gamma Fc receptor agonist	SIRPα/Fc fusion protein antibody	Multiple myeloma	Phase I		NCT05139225	/
					Hematological neoplasm	Phase II	/	NCT02663518	/
TTI-622	TTI-622	Trillium Therapeutics Inc	CD47 antagonist; Immunoglobulin gamma Fc receptor agonist	SIRPα/Fc fusion protein antibody	Acute myelogenous leukemia	Phase I	/	NCT03530683	/
					Multiple myeloma	Phase I	/	NCT03530683, NCT05139225	/
					Multiple myeloma	Phase I	/	NCT03530683	/

**Table 4:** Published results of clinical trials on the use of CD47 antagonists in hematological tumors

Drug Name	NCT Number	Sponsor	Protocol	Indication Detail	Enroll-ment	Age/Race/Healthy Volunteers	Phase	Status	Start Date	Design	Endpoint Types	Efficacy Results	Ref
CC-90002	NCT02641002	Celgene Corp	CC-90002	Subjects With AML and High-Risk MDS	28	Adults (18 and over)	Phase I	Terminted	2016/3/1	Interventional; Treatment; Open Label; Single Group Assignment	Safety; Efficacy; Pharmacokinetics	terminated (preliminary monotherapy data did not offer a sufficiently encouraging profile for further dose escalation/expansion)	[61]
TJ011133	NCT04202003	I-Mab Biopharma Co Ltd	TJ011133	r/r AML/MDS	5	Adults (18–79 years)	Phase I	Recruiting	2020/3/25	Interventional; Treatment; Non-Randomized; Open Label; Single Group Assignment	Efficacy	20% achieved morphologic leukemia-free state	[62]
Hu5F9-G4	NCT03248479	Gilead Sciences Inc	Hu5F9-G4; AZA	untreated AML/MDS	68	Adults (18 and over)	Phase I	Recruiting	2017/9/8	Interventional; Treatment; Non-Randomized; Open Label; Parallel Assignment	Safety; Efficacy; Pharmacokinetics	ORR: 79%; CR/CRi: 56%	[63]
Hu5F9-G4	NCT04435691	MD Anderson Cancer Center	Hu5F9-G4; AZA; VEN	Patients (pts) with Newly Diagnosed AML	23	Adults (18 and over)	Phase I/II	Recruiting	2020/7/28	Interventional; Treatment; Open Label; Single Group Assignment	Safety; Efficacy	CR/CRi: 100%	[64]
Hu5F9-G4	NCT04435691	MD Anderson Cancer Center	Hu5F9-G4; AZA; VEN	Patients (pts) with R/R-post-VEN failure AML	13	Adults (18 and over)	Phase I/II	Recruiting	2020/7/28	Interventional; Treatment; Open Label; Single Group Assignment	Safety; Efficacy	CR/CRi: 27%	[64]
Hu5F9-G4	NCT04435691	MD Anderson Cancer Center	Hu5F9-G4; AZA; VEN	Patients (pts) with R/R-VEN naive AML	8	Adults (18 and over)	Phase I/II	Recruiting	2020/7/28	Interventional; Treatment; Open Label; Single Group Assignment	Safety; Efficacy	CR/CRi: 63%	[64]

### CD47 monoclonal antibodies

#### CC-90002

Although the potential efficacy of CC-90002 in various hematologic malignancies has been demonstrated preclinically [65], a phase I study of patients with R/R AML and MDS (NCT02641002) was terminated due to lack of monotherapy activity and evidence of anti-drug antibodies [61]. In that study, no objective response was observed in any of the enrolled 28 patients (24 patients with AML and 4 with MDS). Of the 15 efficacy-evaluable patients with AML, 14 (93.3%) were classified as having treatment failure; of the 3 efficacy-evaluable patients with MDS, 1 (33.3%) was classified as having treatment failure; the remaining patients achieved the best overall response of SD. Hematologic improvements in MDS were not observed in any of the patient. The reason may be that replacing IgG1 with IgG4 significantly weakened the killing ability of CD47 monoclonal antibodies against tumor cells. At present, the research of CC-90002 in hematological malignancies is not continued.

#### Hu5F9-G4

Preclinical research has demonstrated powerful 5F9 anti-activity in various hematologic malignancies, particularly in AML and MDS [66]. In patients with AML/MDS, 5F9 binds to more than 99% of CD47 receptors in peripheral blood and about 90% in bone marrow [67]. In a clinical trial (NCT03248479), 53% (8/15) untreated AML/MDS patients had a CR or CR with incomplete count recovery (CRi) to 5F9 + azacitidine (AZA) (5/10 in AML and 3/5 in MDS) [63]. In another clinical research (NCT04778397), 56% (10/34) AML patients achieved CR/CRi to 5F9 + AZA [68]. This phase Ib trial also indicated that TP53 mutations were present in 65% of the 34 AML patients examined for efficacy, and that the CR/CRi rate was greater in patients bearing TP53 mutations [68]. Another study included 44 patients (23 newly diagnosed, 8 R/R prior to Venetoclax (VEN)-naïve, and 13 R/R-post-VEN failure). Although the onset of anaemia appears early, it can be alleviated. The CR/CRi rate in the remaining newly diagnosed patients was 100% (15/15), with a CR rate of 87% (13/15). Besides, 7/8 newly diagnosed patients with TP53-mutations were evaluated with a CR/CRi in 100% (7/7) and CR in 86% (6/7). In R/R prior VEN-naïve AML, the CR/CRi rate was 63% (5/8), with median overall survival (OS) not reached (range 1.2–9.7). In R/R prior VEN failure AML the CR/CRi rate was 27% (3/13) with med OS 3.1 (range 0.9–6.5) [64]. Anemia is still the most common AEs of 5F9 during the treatment of hematological tumors. AML patients have decreased hemoglobin and increased blood transfusion demand during 5F9 treatment [69].

In 2019, 5F9 was granted fast-track designation by the FDA for AML/MDS treatment and orphan drug designation by the FDA and European Medicines Agency for AML treatment. However, Gilead Sciences Inc. announced in 2022 that the FDA had placed a partial clinical hold on studies evaluating the combination of 5F9 plus AZA due to an apparent imbalance in investigator-reported suspected unexpected severe adverse reactions between study arms. It is gratifying that the FDA removed the partial clinical hold after reviewing the comprehensive safety data from each trial in 2022. Studies impacted by the action include NCT04313881 (Phase III, MDS), NCT04778397 (Phase III, AML), NCT05079230 (Phase III, MDS), NCT03248479 (Phase Ib, MDS), and NCT04778410 (Phase II, myeloid malignancies and only the AZA combination cohorts). Clinical studies on MDS/AML include NCT03922477 (plus Atezolizumab), NCT04435691 (plus AZA and VEN), NCT04892446(plus daratumumab, plus pomalidomide and dexamethasone, plus bortezomib and dexamethasone). Except for AZA, the combination of 5F9 and other drugs is also undergoing clinical trials. Clinical studies on MDS/AML include NCT03922477 (plus Atezolizumab), NCT04435691 (plus AZA and VEN), NCT04892446 (plus daratumumab, plus pomalidomide and dexamethasone, plus bortezomib and dexamethasone).

#### IBI-188 (Letaplimab)

Studies evaluating the safety and efficacy of IBI-188 in combination with AZA for the treatment of newly diagnosed middle- and high-risk MDS and AML are currently underway in both China and the United States. Notably, IBI-188 in conjunction with AZA for the treatment of hematologic disorders in China is undergoing Phase III clinical trials. Other Phase I trials include Letaplimab in combination with rituximab in advanced lymphoma (NCT03717103), with AZA in AML (NCT04485052), and with AZA in newly diagnosed higher-risk MDS (NCT04485065).

#### TJ011133 (TJC4, Lemzoparlimab)

TJ011133 is now being tested in patients with R/R AML or MDS in a phase I trial (NCT04202003). Four out of five patients had treatment-related AEs. Only one AE was of Grade 3, while the rest were of Grades 1–2. No DLTs or MTDs were detected up to a weekly dose of 10 mg/kg. The average receptor occupancy on peripheral T cells was 74%, 82%, and 84%, respectively, for 1, 3, and 10 mg/kg. One main refractory AML patient attained morphologic leukemia-free status following two cycles of 1 mg/kg TJ011133 therapy [62]. TJ011133 in combination with AZA has been approved for a phase III clinical trial in the treatment of primary higher risk MDS.

## Challenges and future perspectives of CD47/SIRPα immune checkpoint for tumors

### Challenges

CD47 antagonists have shown some promise results in preclinical and clinical studies for treating hematologic tumors and lymphomas. For the foreseeable future, CD47/SIRPα will one day become an equally promising immunotherapy as PD-1/PD-L1. In order to reach the full potential of CD47/SIRPα immune checkpoint-based immunotherapy, further studies are necessary. Currently, Hu5F9-G4, ALX-148 and TJ011133 are undergoing Phase III clinical studies, and it is anticipated that these medications will be the first to file marketing applications for new drugs.

However, the development of CD47 monoclonal antibodies still faces three major challenges: therapeutic effectiveness, safety concerns, and lack of published data. CD47 is widely expressed in normal cells, this implies that substantial dosages or frequent administration may be necessary to achieve effective therapeutic CD47 blockage. Preclinical studies have shown that 40–60% CD47 receptor occupancy is required for the induction of phagocytosis [51]. However, extra caution is required given that large doses or frequent administration may result in treatment-related adverse effects. Besides, when tumor cells express both SIRPα and TSP-1 which inhibit the CD47-SIRPα interaction [70], the effective dose of CD47 antagonist may need to be modified. Additionally, sufficient macrophage activation requires triggering of Fc receptors, hence proper human IgG should be chosen [17]. Even though human IgG1 works better to stimulate macrophages, it also causes immune cells to attack RBCs. Therefore, most companies have chosen to develop human IgG4-type antibodies, which significantly lower the antitumor activity [71]. Moreover, CD47-targeted bispecific antibodies only act on particular tumor types, therefore malignancies must be identified and classified to determine the most effective treatment. So far, most clinical trials on CD47 antagonists are in phase I and II, implying that optimal dosage of dual antibodies requires further investigation.

Hematotoxicity, particularly anemia, is the most frequent adverse effect of CD47 inhibitors. Even if there is evidence of promising antitumor efficacy, CD47 antagonists are associated with anemia since RBCs express a substantial amount of CD47. Additionally, activating certain epitopes on the Ig variable structural region of CD47 has been reported to induce fast T cell apoptosis and depletion [72]. Application of CD47 antagonists may also cause other safety issues, for example, unexpected immunological problems may occur as most immune cells express CD47; inhibiting SIRPα may produce nervous system malfunction, such as aberrant synaptic pruning in microglia neurodegeneration, since SIRPα is highly expressed in central and peripheral nervous system cells [13, 73]. Finally, due to the sequence similarities of SIRP family, CD47 may cross-react with other SIRPs, resulting in unintended side effects. CD47 has been found to bind to SIRPγ and positively regulate human T cell activation and proliferation [5]. Use of CD47 inhibitors may result in T-cell function suppression, which deserves further investigation in the future.

Last but not least, there is a lack of published data on the affinity of CD47 antagonists for checkpoint binding, either with pure CD47 and SIRPα protein or on appropriate human cells, hindering more insightful analysis on this topic.

### Future perspectives

**Fig. 4:**
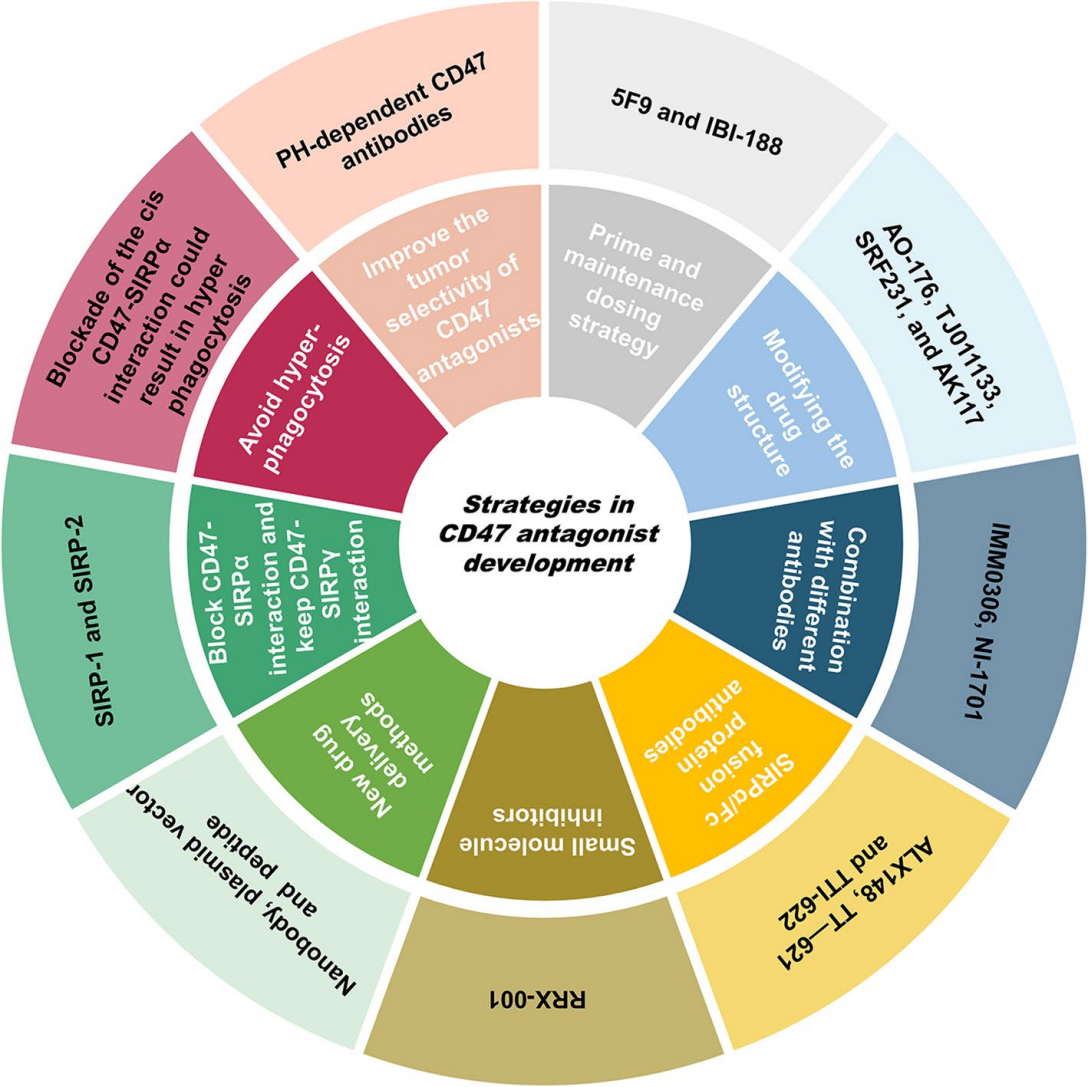
Future strategies for developing CD47 antagonists**.** Abbreviation: CD47, cluster of differentiation 47; SIRP, signal-regulatory protein

In order to increase the efficacy and safety of CD47 antagonists, the following strategies of antagonist development may emerge in the future (Fig. 4): (1) Using the strategy of CD47 antagonist prime and maintenance dosing (*e.g*., 5F9 and IBI-188); (2) Modifying the drug structure of CD47 monoclonal antibody *(e.g*., AO-176, TJ011133, SRF231, and AK117); (3) Fusion protein of CD47/SIRPa combining with different antibodies, such as CD20, CD19, could be of a promising strategy in the immunotherapy targeting CD47/SIRPa axis (*e.g.*, IMM0306, NI-1701) [17, 74]; (4) Development of SIRPα/Fc fusion protein antibodies; (5) Development of small-molecule inhibitors (*e.g*., RRX-001, QPCTL antibodies); (6) Introduction of new drug delivery methods (*e.g*., CD47 nanobody [55], plasmid vector [75], and CD47/SIRPα blocking peptide [76]); (7) Since binding of CD47 to SIRPγ enables T cell activation and proliferation [5], blockade of SIRPα-CD47 interaction while preserving SIRPγ binding to CD47 may be a strategy for cancer immunotherapy (*e.g.*, SIRP-1 and SIRP-2) [77]; (8) SIRPα engages with CD47 in either *cis* or *trans* behavior in different scenarios. SIRPα expressed in macrophages exhibits *trans* binding to CD47 that are expressed in other types of ‘self’ cells, leading to local SIRPα accumulation and inhibition of ‘self’cell engulfment including the tumor cells [35, 78]. On the other hand, CD47 expressed on macrophages has the potential to modulate phagocytosis through a *cis* interaction with SIRPα that is also expressed on macrophages. Blockade of the *cis* CD47-SIRPα interaction could result in hyper phagocytosis [79]. Therefore, extra consideration needs to be taken in the development of CD47 antagonists as they may lead to different clinical outcomes; (9) Improvement of the tumor selectivity of CD47 antagonists may also be a future strategy (*e.g.,* a PH-dependent CD47 antibody) [80].

In conclusion, given that CD47 antagonists enable cancer cells to escape macrophage-mediated phagocytosis, inhibiting the CD47/SIRPα axis is a potential cancer treatment strategy. A deeper understanding of the mechanisms and processes by which tumor cells avoid immune clearance and improving CD47 antagonist administration routes would contribute to developing effective and safe antitumor medicines. The latest clinical research advances and detailed information were presented in tables to aid the readers for quick search of the contents of interest.

## Data Availability

Not applicable.

## Abbreviations

ADCC: Antibody-dependent cell-mediated cytotoxicity
AE: Adverse event
ALL: Acute lymphoblastic leukemia
AML: Acute myeloid leukemia
AZA: Azacitidine
CAILS: Composite assessment of index lesion severity
CD: Cluster of differentiation
CDC: Complement-dependent cytotoxicity
CLL: Chronic lymphocytic leukemia
CR: Complete response rate
CRi: CR with incomplete blood count recovery
CTR: The China drug trials registry
DLBCL: Diffuse large B cell lymphoma
DLT: Dose-limiting toxicity
FDA: Food and Drug Administration
FIH: First-in-human
FL: Follicular lymphoma
ITIM: Immunoreceptor tyrosine-based inhibitor motif
MDS: Myelodysplastic syndrome
MM: Multiple myeloma
MTD: Maximum tolerated dose
NCT: The US national clinical trials registry
NHL: Non-Hodgkin lymphoma
NHP: Non-human primate
ORR: Overall response rate
OS: Overall survival
PD-1: Programmed cell death receptor-1
PD-L1: Programmed cell death ligand-1
PR: Partial response
QPCTL: Glutaminyl-peptide cyclotransferase-like
QW: Every week
R/R: Relapsed/refractory
RBC: Red blood cells
SD: Stable disease
SHP: Src homology phosphatase
SIRP: Signal regulatory protein
SIRPα: Signal regulatory protein alpha
TSP-1: Thrombospondin-1
VEN: Venetoclax
